# Oxaliplatin-induced loss of phosphorylated heavy neurofilament subunit neuronal immunoreactivity in rat DRG tissue

**DOI:** 10.1186/1744-8069-5-66

**Published:** 2009-11-18

**Authors:** Stephen MF Jamieson, Joshuan Subramaniam, Johnson J Liu, Nancy N Jong, Virginia Ip, Bronwen Connor, Mark J McKeage

**Affiliations:** 1 Department of Pharmacology and Clinical Pharmacology, School of Medical Sciences, Faculty of Medical and Health Sciences, The University of Auckland, Auckland, New Zealand

## Abstract

**Background:**

Oxaliplatin and related chemotherapeutic drugs cause painful chronic peripheral neuropathies in cancer patients. We investigated changes in neuronal size profiles and neurofilament immunoreactivity in L5 dorsal root ganglion (DRG) tissue of adult female Wistar rats after multiple-dose treatment with oxaliplatin, cisplatin, carboplatin or paclitaxel.

**Results:**

After treatment with oxaliplatin, phosphorylated neurofilament heavy subunit (pNF-H) immunoreactivity was reduced in neuronal cell bodies, but unchanged in nerve fibres, of the L5 DRG. Morphometric analysis confirmed significant changes in the number (-75%; *P *< 0.0002) and size (-45%; *P *< 0.0001) of pNF-H-immunoreactive neurons after oxaliplatin treatment. pNF-H-immunoreactive neurons had overlapping size profiles and co-localisation with neurons displaying cell body immunoreactivity for parvalbumin, non-phospho-specific neurofilament medium subunit (NF-M) and non-phospho-specific neurofilament heavy subunit (NF-H), in control DRG. However, there were no significant changes in the numbers of neurons with immunoreactivity for parvalbumin (4.6%, *P *= 0.82), NF-M (-1%, *P *= 0.96) or NF-H (0%; *P *= 0.93) after oxaliplatin treatment, although the sizes of parvalbumin (-29%, *P *= 0.047), NF-M (-11%, *P *= 0.038) and NF-H (-28%; *P *= 0.0033) immunoreactive neurons were reduced. In an independent comparison of different chemotherapeutic agents, the number of pNF-H-immunoreactive neurons was significantly altered by oxaliplatin (-77.2%; *P *< 0.0001) and cisplatin (-35.2%; *P *= 0.03) but not by carboplatin or paclitaxel, and their mean cell body area was significantly changed by oxaliplatin (-31.1%; *P *= 0.008) but not by cisplatin, carboplatin or paclitaxel.

**Conclusion:**

This study has demonstrated a specific pattern of loss of pNF-H immunoreactivity in rat DRG tissue that corresponds with the relative neurotoxicity of oxaliplatin, cisplatin and carboplatin. Loss of pNF-H may be mechanistically linked to oxaliplatin-induced neuronal atrophy, and serves as a readily measureable endpoint of its neurotoxicity in the rat model.

## Background

Oxaliplatin is a platinum-based chemotherapeutic agent approved for the treatment of colorectal cancer [1]. Although particularly effective for treating colorectal cancer, oxaliplatin causes neurotoxicity in a high percentage of patients [2] that is dose-limiting and can only be prevented by reducing or stopping the drug. Oxaliplatin causes acute and chronic forms of neurotoxicity in the clinic. Acute oxaliplatin neurotoxicity presents with neuro-sensory symptoms that develop during or soon after each drug infusion then recover within a few days or weeks [2,3]. These symptoms are exacerbated by cold exposure and associated with electrophysiological signs of peripheral nerve hyperexcitability [4]. With repeated treatment, oxaliplatin causes a chronic sensory neuropathy with distal paraesthesiae and dyesthesiae, loss of tendon reflexes, vibration sense and proprioception, and sensory ataxia in severe cases [2,3]. The chronic neurotoxicity of oxaliplatin is cumulative and less reversible than its acute syndrome.

There have been previous studies of oxaliplatin-induced neurotoxicity in rodent models. Single doses of oxaliplatin have been reported to acutely disturb nucleolar morphology in DRG neurons [5] and alter behavioural responses indicating sensory allodynia and hyperalgesia [6,7]. Chronic oxaliplatin treatment causes reduced sensory nerve conduction in the tail or hind-limb of treated rodents [8,9], altered sensory responses [10,11] and changes in the size profiles of DRG neurons [8,9,12] suggestive of neuronal atrophy or the loss of DRG neurons. The doses of oxaliplatin employed in these previous rodent studies have varied widely but were often lower than those used clinically, when expressed as per unit of body surface area or considered on the basis of relative systemic exposure achieved in rats [13] and humans [14].

In the current study, we investigated the effect of oxaliplatin on neuronal size profiles and neurofilament immunoreactivity in DRG tissue from adult Wistar rats following multiple treatments to a cumulative dose of approximately 180 mg/m^2^. This dose was broadly comparable to those that are achieved clinically [1] and induce changes in sensory nerve conduction and DRG morphology in the rat model [8]. Immunohistochemistry was used to identify subpopulations of DRG neurons and assess their relative susceptibilities to oxaliplatin-induced neurotoxicity, as in recent studies [8,15]. The RT97 primary antibody employed in these studies recognises phosphorylated KSP repeats in the tail domain of phosphorylated neurofilament heavy subunit (pNF-H) [16]. The epitopes of the RT97 antibody are strongly expressed in rat DRG tissue within the cell bodies of subpopulations of large DRG neurons and large-diameter myelinated nerve fibres [17]. Phosphorylated neurofilaments are major cytoskeletal proteins of large myelinated sensory neurons [18]. Disturbance of neurofilament phosphorylation has been implicated in a wide range of neurodegenerative diseases [19] but its role in oxaliplatin-induced neurotoxicity is unknown.

In this paper, we report that neuronal pNF-H expression, as determined by RT97 immunohistochemistry of rat DRG tissue, was significantly reduced after oxaliplatin treatment. This loss of pNF-H immunoreactivity was shown to correspond with the relative neurotoxicity of oxaliplatin, cisplatin and carboplatin, but was not associated with the loss of DRG cells, generalised reduction of neuronal marker or neurofilament expression, or with paclitaxel-induced neurotoxicity.

## Results

### Oxaliplatin-induces loss of neuronal pNF-H immunoreactivity

**Figure 1:**
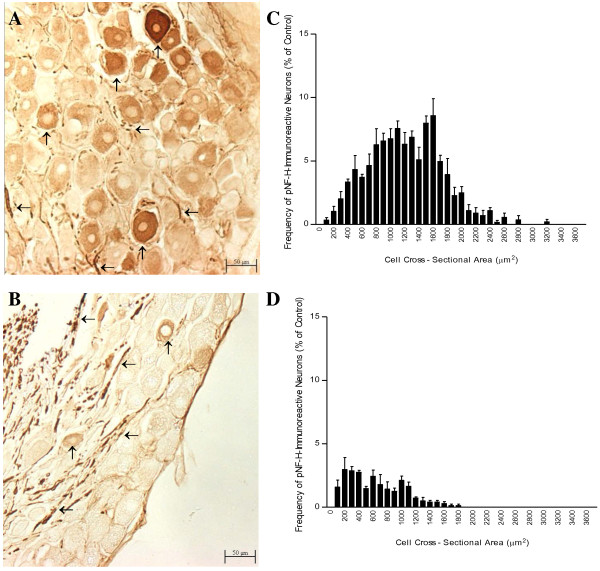
**Loss of neuronal pNF-H immunoreactivity induced by oxaliplatin in rat DRG tissue**. Photomicrographs (A, B; magnification: 100×) and cell body size frequency histograms (C, D) of pNF-H-immunoreactive L5 DRG neurons from rats treated with the control vehicle (A, C) or oxaliplatin (B, D). Oxaliplatin reduced pNF-H immunostaining of neuronal cell bodies (↑) without changing pNF-H immunoreactivity of ganglionic nerve fibers (←). Cell body size frequency histograms of pNF-H-immunoreactive neurons were altered by oxaliplatin. Bars represent the mean and standard error of the mean for 4 animals.

**Figure 2:**
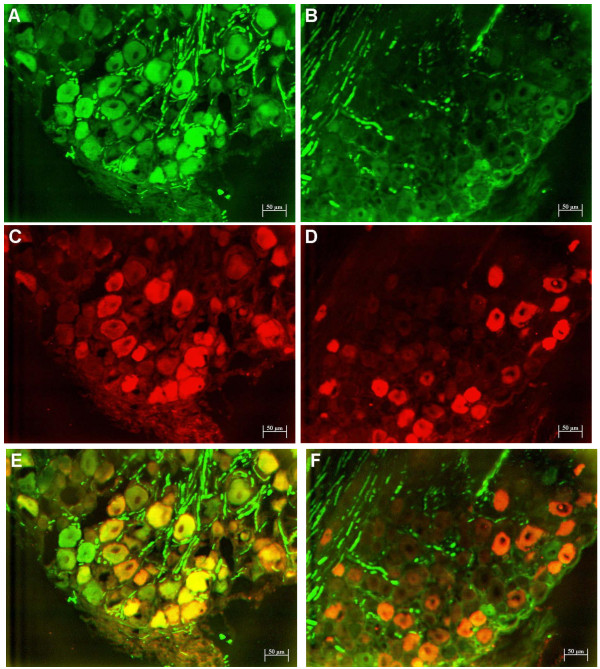
**Parvalbumin and pNF-H fluorescent double immunolabelling of rat DRG tissue from control and oxaliplatin-treated animals**. Representative DRG sections displaying fluorescent immunolabelling of pNF-H (FITC; green) (A, B) and parvalbumin (Cy3; red) alone (C, D) and overlaid to determine the extent of co-expression (yellow) (E, F) in L5 DRG of animals treated with the control vehicle (A, C, E) or oxaliplatin (B, D, F). Oxaliplatin reduced pNF-H cell body staining and its co-localisation with parvalbumin. The cell bodies of immunoreactive neurons appeared smaller after oxaliplatin. The number of nerve fibres staining for pNF-H and cell bodies staining for parvalbumin appeared unchanged after oxaliplatin. Magnification = 100×.

After the treatment of rats with oxaliplatin, pNF-H immunoreactivity was reduced in neuronal cell bodies, but appeared unchanged in nerve fibres, of the L5 DRG (Fig 1 and 2). pNF-H immunohistochemistry was carried out using the RT97 primary antibody on cryosections of L5 DRG from animals treated with oxaliplatin or 5% glucose (vehicle control). In control DRG (Figure 1A and 2A), pNF-H immunoreactivity was associated with ganglionic nerve fibres and large neuronal cell bodies, with cytoplasmic staining and nuclei sparing, consistent with a previous report [17]. After treatment with oxaliplatin twice weekly for 8 weeks at a maximum tolerated dose (1.85 mg/kg/dose), pNF-H immunoreactivity of nerve fibres appeared to be relatively unchanged but that of the DRG cell bodies was greatly reduced (Figure 1B and 2B). Cell body size frequency histograms of pNF-H immunoreactive neurons were markedly altered by oxaliplatin treatment (Figure 1C, D).

Morphometric analysis confirmed that oxaliplatin treatment was associated with statistically significant reductions in the number and size of pNF-H immunoreactive neurons (Table 1). Defined as DRG neurons with cell body staining greater than background staining of a negative control, pNF-H immunoreactive neurons accounted for approximately 20% of the overall population of DRG neurons in control animals versus about 5% in animals treated with oxaliplatin (-75%; *P *< 0.0002). In addition, the size of pNF-H-immunoreactive neurons was altered by oxaliplatin treatment as evident from significant changes in mean cell body size (-45%, *P *< 0.0001), and in the percentages of neurons with large (>1750 μm^2^) (-95%; *P *< 0.001), medium (750-1750 μm^2^) (-41%; *P *< 0.0005) or small cell bodies (<750 μm^2^) (+247%; *P *< 0.0001).

**Table 1:** Effect of oxaliplatin on morphometry of L5 DRG neurons with immunoreactivity for pNF-H, parvalbumin, NF-M or NF-H.

		Frequency of Immunoreactive Cells (%)	Mean Cell Body Area of Immunoreactive Cells (μm^2^)	Frequency of Small Cells (%)	Frequency of Medium Cells (%)	Frequency of Large Cells (%)
pNF-H	Control	21.0 ± 1.9	1259 ± 36	17.2 ± 1.7	67.2 ± 1.8	15.6 ± 2.3
	Oxaliplatin	5.2 ± 0.3	696 ± 47	59.7 ± 3.8	39.5 ± 3.3	0.82 ± 0.5
	Percent change	-75%	-45%	247%	-41%	-95%
	*P*	<0.0002	<0.0001	<0.0001	<0.0005	<0.001

Parvalbumin	Control	17.4 ± 1.9	1042 ± 31	40.2 ± 1.9	44.6 ± 1.8	15.5 ± 2.9
	Oxaliplatin	18.2 ± 4.2	739 ± 75	59.3 ± 12	37.2 ± 5.7	2.9 ± 2.2
	Percent change	4.6%	-29%	49%	-17%	-81%
	*P*	0.82	0.047	0.09	0.35	0.008

NF-M	Control	41.7 ± 4.0	997 ± 29	32.2 ± 2.2	63.6 ± 3.3	4.2 ± 1.6
	Oxaliplatin	41.4 ± 4.6	888 ± 21	39.4 ± 4.5	59.0 ± 4.8	1.6 ± 0.3
	Percent change	-1%	-11%	22%	-7%	-62%
	*P*	0.96	0.038	0.22	0.47	0.19

NF-H	Control	96.0 ± 0.9	1068 ± 42	38.8 ± 6.8	46.0 ± 7.4	15.2 ± 0.8
	Oxaliplatin	95.9 ± 1.0	765 ± 24	58.9 ± 1.8	37.4 ± 1.4	3.6 ± 0.6
	Percent change	0%	-28%	52%	-19%	-76%
	*P*	0.93	0.0033	0.045	0.32	<0.001

Animals were treated with oxaliplatin or drug vehicle alone (control group) twice per week for 8 weeks. Values represent the mean and standard error of the mean for 4 to 5 animals/group.

### Neuronal parvalbumin and non-phospho-specific neurofilament immunoreactivity persists following oxaliplatin treatment

Next we examined the effect of oxaliplatin on the expression of parvalbumin, a marker of large DRG neurons. As previously reported [8,20,21], parvalbumin immunoreactivity in control DRG (Fig 2C) was localized to the cell bodies of a subset of neurons, whose size profile (Table 1) was similar to the neuronal subpopulation defined by cell body immunoreactivity for pNF-H. Double immunofluorescence labeling confirmed extensive co-localization of parvalbumin and pNF-H immunoreactivity in the neuronal cell bodies of control DRG (Figure 2E). After oxaliplatin treatment, parvalbumin immunoreactivity persisted (Fig 2D) but its co-localization with pNF-H was reduced (Fig 2F) due to loss of cell body staining of pNF-H (Fig 2B). Morphometric analysis confirmed that the number of parvalbumin-immunoreactive neurons was not markedly changed (4.6%; NS) by oxaliplatin treatment, but their size was reduced as evident from significant changes in their mean cell body area (-29%; *P *= 0.047) and in the percentage of large neurons (>1750 μm^2^) (-81%; *P *= 0.008).

**Figure 3:**
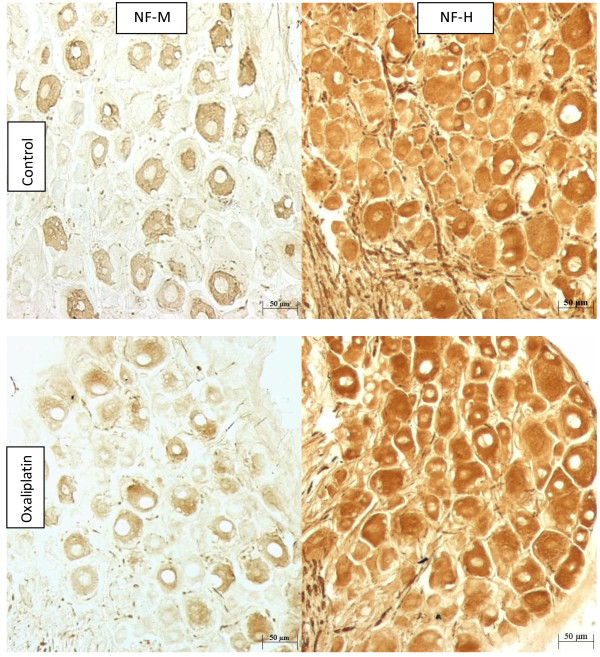
**Non-phospho-specific neurofilament immunoreactivity of rat DRG tissue**. Immunohistochemistry using non-phospho-specific primary antibodies for NF-M and NF-H in representative sections of DRG from control and oxaliplatin treated animals. Oxaliplatin did not change the numbers of immunoreactive neurons but reduced their size.

Different primary antineurofilament antibodies are known to vary in their patterns of immunostaining of DRG due to variation in their affinities for different neurofilament subunits and neurofilament phosphorylation states [22]. This provided an opportunity to investigate whether the loss of neuronal RT97 immunoreactivity was due to a generalised reduction in neurofilament expression in DRG neurons induced by oxaliplatin. Immunostaining with non-phospho-specific antibodies for neurofilament medium subunit (NF-M) and neurofilament heavy subunit (NF-H) showed no changes in numbers of immunoreactive neurons, but did show reductions in their size profiles, after treatment with oxaliplatin (Figure 3, Table 1). Non-phospho-specific NF-M immunoreactivity was present in cell bodies of small, medium and large DRG neurons (Figure 3) accounting for approximately 40% of the overall neuronal population in L5 DRG from both control and oxaliplatin-treated animals (-1%; *P *= 0.96). However, the mean cell body size of NF-M immunoreactive neurons was significantly altered by oxaliplatin (-11%; *P *= 0.038). Non-phospho-specific NF-H immunoreactivity was present in nerve fibres and many neuronal cell bodies of all sizes (Figure 3) accounting for >95% of the overall population of DRG neurons in both control and oxaliplatin-treated animals (0%; *P *= 0.93). Consistent with previous reports, relatively intense NF-H staining was seen in a neuronal subpopulation with large cell bodies, which are known for their high neurofilament content and myelinated A-type nerve fibres [18]. Although there was no change in the numbers of NF-H-immunoreactive neurons, their size was significantly altered by oxaliplatin as evident by changes in their mean cell body area (-28%; *P *= 0.0033), and in the percentages of large (>1750 μm^2^) (-76%; *P *= 0.0003) or small neurons (<750 μm^2^) (+52%; *P *< 0.05).

### Comparative effect of chemotherapeutic drugs on neuronal pNF-H immunoreactivity

**Figure 4:**
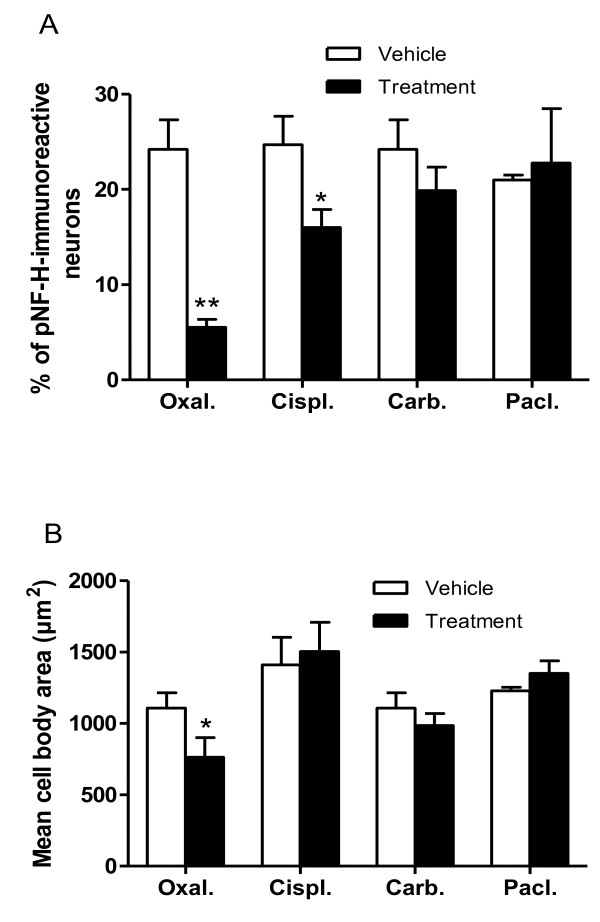
**Comparative effect of anticancer drugs on neuronal pNF-H immunoreactivity**. DRG were analysed after multiple-dose treatment with maximally tolerated doses of oxaliplatin (Oxal; 1.85 mg/kg/dose twice per week for 8 weeks), cisplatin (Cispl; 1 mg/kg/dose twice per week per 8 weeks), carboplatin (Carb; 8 mg/kg/dose twice per week per 8 weeks) and paclitaxel (Pacl; 12.5 mg/kg/dose once per week for 9 weeks). Bars represent the mean and standard deviation of 3 to 6 animals, * P < 0.01.

Finally we compared the effect of different chemotherapeutic agents on neuronal pNF-H immunoreactivity in rat DRG tissue. Groups of animals were treated for 8 weeks with multiple-doses of oxaliplatin (1.85 mg/kg/dose twice per week), cisplatin (1 mg/kg/dose twice per week) or carboplatin (8 mg/kg/dose twice per week), or for 9 weeks with multiple-doses of paclitaxel (12.5 mg/kg/dose once per week), or their respective drug vehicles as matching control groups. These doses of oxaliplatin [8,23,24], cisplatin [24], carboplatin [24] and paclitaxel [25] were previously shown to alter sensory nerve conduction and DRG neuronal morphometric parameters in this rat model in keeping with the induction of a peripheral neuropathy. There was no mortality during the treatment period but the amount of body weight gained during the experiment was less in the treatment groups (range; 5 to 17% of baseline) than in the control groups (range; 20 to 27% of baseline). The effect of oxaliplatin on the number and size of pNF-H-immunoreactive neurons was confirmed in this independent experiment. After treatment, the number of pNF-H-immunoreactive neurons was significantly altered by oxaliplatin (-77.2%; *P *< 0.0001) and cisplatin (-35.2%; *P *= 0.003) but not by carboplatin or paclitaxel (Figure 4A). The mean cell body area of pNF-H-positive neurons was significantly changed by oxaliplatin (-31.1%; *P *= 0.008) but not by cisplatin, carboplatin or paclitaxel (Figure 4B).

## Discussion

In this paper we report, for the first time, an effect of oxaliplatin on neurofilament expression in rat DRG tissue as determined by immunohistochemistry using the RT97 primary anti-neurofilament antibody. The epitopes of the RT97 antibody are phosphorylated KSP repeats within the tail domain of phosphorylated neurofilament heavy subunit [16], which are expressed in a specific pattern in DRG tissue of healthy adult rats [17]. In DRG these epitopes are expressed within the cell bodies of a subpopulation of large neurons, presumably during early post-translational modification of neurofilament subunits, and in their axons, where neurofilament subunits become hyper-phosphorylated during their polymerisation into stabilised polymeric complexes [26]. Aberrant neurofilament phosphorylation, as indicated by altered tissue immunoreactivity for phospho-specific anti-neurofilament primary antibodies, such as the RT97 antibody, has been associated with other disorders of DRG neurons [27,28], but a role in oxaliplatin neurotoxicity has not been considered.

We demonstrated that chronic oxaliplatin treatment was associated with a specific pattern of loss of pNF-H immunoreactivity in rat DRG tissue in the current study. The loss of pNF-H immunoreactivity was evident visually from qualitative changes in the intensity of neuronal cell body immunostaining in DRG sections and by statistically significant reductions in the numbers and size of pNF-H-immunoreactive neurons in oxaliplatin-treated animals, confirmed in two independent experiments. Strong pNF-H immunoreactivity appeared to remain in DRG nerve fibres after oxaliplatin treatment indicating that its loss was specific for the neuronal cell bodies, and that the treatment caused no nonspecific masking of pNF-H epitopes, under these experimental conditions. In control DRG, the neuronal immunoreactivity for pNF-H overlapped or colocalised with parvalbumin, non-phospho-specific-NF-M and non-phospho-specific-NF-H, but the number of DRG neurons displaying immunoreactivity for these primary antibodies was not changed by oxaliplatin. Therefore, the loss of neuronal pNF-H immunoreactivity was not associated with the loss of DRG cells or any generalised reduction of neuronal marker or neurofilament expression induced by oxaliplatin.

The present study also demonstrated that the extent of loss of pNF-H immunoreactivity corresponded to the relative neurotoxicity of oxaliplatin, cisplatin and carboplatin. When these platinum agents were ranked according to their effect on the number of pNF-H-immunoreactive DRG neurons, oxaliplatin had the greatest effect, followed by cisplatin and then carboplatin had the least. Their ranking corresponded with the relative cumulative dose-potencies of oxaliplatin, cisplatin and carboplatin for reducing sensory nerve conduction velocity in rats, which occurs after cumulative doses of 15, 46.7 and 302 μmol/kg, respectively [24]. In addition, this ranking corresponded with the proportion of patients developing peripheral neurotoxicity of any severity grade after treatment with these platinum drugs, which is reported to occur in ~90% [29], ~50% [30] and ~6% [31] of patients treated with oxaliplatin, cisplatin and carboplatin, respectively.

These findings link the loss of pNF-H with the neurotoxicity of oxaliplatin, although its exact role in this toxicity remains to be elucidated. Phosphorylated neurofilaments have important physiological roles in maintaining axonal calibre and fast conduction velocity of large myelinated nerve fibres [18,26], and their loss causes neuronal and axonal atrophy, and reduced sensory nerve conduction velocity, of DRG neurons [32-34]. Therefore, the loss of neuronal pNF-H expression demonstrated in the current study may be causally linked to the decreased size profiles of DRG neurons and reduced sensory nerve conduction velocity, which are induced by chronic oxaliplatin treatment in rodent models [8,9,12].

The molecular mechanisms responsible for loss of pNF-H immunoreactivity induced by oxaliplatin are unclear and require further study. Defects in early neurofilament phosphorylation could account for the loss of RT97 cell body staining without changes in its nerve fibre immunoreactivity or altered immunoreactivity of non-phospho-specific antineurofilament primary antibodies. The main pharmacological mechanism of platinum-based drugs is the formation of platinum-DNA adducts that inhibit DNA replication and transcription [35]. After exposure to platinum drugs, DNA-platinum adducts have been detected in DRG neurons [36,37] and their level is correlated with the severity of neurotoxicity [38,39]. Therefore, the loss of RT97 immunoreactivity could occur due to inhibited transcription of neurofilament kinase genes. However, confirmation of a mechanism involving defective neurofilament phosphorylation or inhibited transcription would be technically difficult in DRG tissue because of the confounding effects of persisting RT97 immunoreactivity of the ganglionic nerve fibres and non-specific inhibition of DNA transcription by platinum drugs [40]. Whatever the mechanism, it was evident from this study that pNF-H is a specific marker of DRG neuronal subpopulations particularly susceptible to damage from chronic oxaliplatin exposure, and changes in numbers of pNF-H immunoreactive neurons, are readily measureable endpoints of oxaliplatin neurotoxicity in the rat. Similarly, the current study confirmed our previous observations [8] of parvalbumin being a specific marker of DRG neurons susceptible to oxaliplatin toxicity and significant changes in size profiles of parvalbumin immunoreactive neurons during this neurotoxicity. Detecting oxaliplatin-induced neurotoxicity in the rat model using pNF-H or parvalbumin immunohistochemistry is statistically more powerful and utilises fewer animals than nerve conduction studies. However, unlike immunochemical endpoints, nerve conduction measurements can be repeated at different times in the same animal and provide functional information.

Paclitaxel causes peripheral neurotoxicity in a high proportion of treated patients [41] and reductions in sensory nerve conduction velocity in the rat [25,42-44], but had no effect on the number or size of pNF-H immunoreactive neurons in this study. The mechanism of paclitaxel neurotoxicity may involve microtubule binding and disturbance of microtubule polymerisation with resulting axonal damage [43,45] and secondary reactive changes in DRG cell bodies [25,46,47]. In contrast, the mechanism of oxaliplatin neurotoxicity may involve a loss of phosphorylated neurofilaments at the level of DRG cell bodies with secondary changes in axonal calibre and conduction velocity. In this way, disturbance of major neuronal cytoskeletal proteins, such as microtubules and neurofilaments, may be a common mechanistic theme whereby different anticancer drugs from various classes damage the peripheral nervous system.

## Conclusion

In conclusion, this study has demonstrated a specific pattern of loss of pNF-H immunoreactivity in rat DRG tissue that corresponds with the relative neurotoxicity of oxaliplatin, cisplatin and carboplatin. Loss of pNF-H may be mechanistically linked to oxaliplatin-induced neuronal atrophy and serves as readily measureable endpoints of oxaliplatin neurotoxicity in the rat model.

## Methods

### Animals and Drugs

Age-matched 10-week old female Wistar rats were used for experiments that weighed approximately 270 g at the commencement of the study. All animals were housed in a temperature and humidity-controlled environment with uninhibited access to food and water. Oxaliplatin (Sigma-Aldrich, St. Louis, MO, USA and Sanofi-Synthelabo NZ Ltd, Auckland, NZ) and carboplatin (Mayne Pharma, Vic, Australia) were diluted for injection in 5% dextrose (Baxter Healthcare, Old Toongabbie, Australia) for intraperitoneal injection at 15 ml/kg. Cisplatin (Sigma) was diluted in 0.9% sodium chloride (Baxter Healthcare) for intraperitoneal injection at 15 ml/kg. Paclitaxel (Phytogen Life Sciences Inc., Delta, BC, Canada) was solubilised in a 1:1 solution of Cremophor EL (Sigma-Aldrich) and ethanol to make a stock solution of 6 mg/ml, then further diluted with 0.9% NaCl (Baxter Healthcare) for administration by intraperitoneal injection at an injection volume of 12.5 ml/kg. Animals were treated twice per week either with oxaliplatin (1.85 mg/kg), carboplatin (8 mg/kg) or their control drug vehicle of 5% dextrose, or with cisplatin (1 mg/kg) or its control vehicle of 0.9% sodium chloride, an injection volume of 15 ml/kg for 8 weeks. Paclitaxel-treated animals received 12.5 mg/kg of drug once weekly for a total of 9 weeks, and control animals were treated with the Cremophor EL/ethanol/0.9% NaCl solution at the same dosing frequency and injection volume. To prevent time-dependent variation in pharmacokinetics and pharmacodynamics all injections were performed between 1 and 3 p.m. The Animal Ethics Committee of the University of Auckland approved all animal procedures.

### Single-label Immunohistochemistry

One week after the conclusion of treatment, terminal anaesthesia was induced by administering 0.9 ml of 3 mg/ml pentobarbitone (Chemstock Animal Health Ltd, Christchurch, New Zealand). Subsequently, transcardiac perfusion with 60 ml of 0.9% NaCl (Baxter Healthcare) followed by 60 ml of 4% paraformaldehyde in 0.1 M phosphate buffer was carried out. L5 DRGs were carefully dissected from each animal, post-fixed in 4% paraformaldehyde for 2-6 hours and cryoprotected in a 30% sucrose solution until the tissues sunk. Following cryoprotection, the DRG were placed in Tissue-Tek OCT compound (Sakura Finetek, Torrance, CA, USA), snap frozen in liquid nitrogen and stored at -80°C. Each dorsal root ganglion was sectioned on a cryostat (Leica CM 3050) at a thickness of 10 μm onto polylysine-coated slides that were then stored at -80°C. For immunostaining, frozen tissue slides were warmed to room temperature, washed in PBS containing 0.2% Triton X-100 and incubated in 1% H_2_O_2 _in 50% methanol for 10 minutes. To prevent non-specific binding, the slides were blocked for 1 hour in PBS containing 0.2% Triton X-100 with 3% normal goat serum (Sigma-Aldrich) and 20 mg/ml bovine serum albumin (Sigma-Aldrich). Next, the slides were incubated overnight in a humidity chamber with either the mouse monoclonal antibody to the phospho-specific NF-H subunit (RT-97 clone; 1:100; CBL212, Chemicon International, Temecula, CA, USA), rabbit polyclonal anti-parvalbumin (PVA3) primary antibody (1:1000; P. Emson, Cambridge, UK), rabbit polyclonal non-phospho-specific antineurofilament 200 IgG fraction (1:1000; Sigma N4142) or mouse monoclonal non-phospho-specific antineurofilament medium subunit (1:1000; Sigma N5264). The slides were rewashed and incubated with either an anti-mouse or anti-rabbit biotinylated secondary antibody (1:500; Sigma-Aldrich) for 2.5 hours. After further washes, the slides were incubated for 3 hours in an extravidin-peroxidase conjugate (1:500; Sigma-Aldrich). Staining was visualised with 0.5 mg/ml 3,3'-diaminobenzidine tetrahydrochloride (AppliChem, Darmstadt, Germany) and 0.01% H_2_O_2 _in 0.4 M phosphate buffer for 10 minutes. Finally, the slides were washed, dehydrated through a series of alcohols, cleared in xylene and coverslipped. The DRG sections were analysed by light microscopy with digital images generated by an Axiocam camera (Carl Zeiss Vision, Hallbergmoos, Germany) and quantitative analysis performed using AxioVision 3.0 (Carl Zeiss Software) software. The cross-sectional area was measured for each immunoreactive neuron and the frequency of expression was generated by counting every cell and expressing the count of immunoreactive neurons as a percentage of the total cell count. Immunoreactive DRG neurons were also categorised on the basis of size into small (cross-sectional area <750 μm^2^), medium (750-1750 μm^2^) and large (>1750 μm^2^) sized cells.

### Fluorescent Double Labelling Immunohistochemistry

Frozen DRG slides were defrosted, washed, incubated in H_2_O_2 _with methanol and blocked as described previously for single-label immunohistochemistry. Each slide was incubated overnight in a humidity chamber in both mouse anti-pNF-H (1:100) and rabbit anti-parvalbumin (1:1000) primary antibodies. The slides were then washed and incubated in the dark for 4 hours in both anti-rabbit cy3 (1:200; Jackson Laboratories, West Grove, PA, USA) and biotinylated anti-mouse secondary antibodies (1:200, Sigma). The slides were rewashed and incubated in the dark for 3 hours in FITC tertiary antibody (1:200; Sigma-Aldrich). Finally, the slides were washed, cover slipped with Citifluor (Agar Scientific, Essex, UK) and stored overnight at 4°C to prevent bleaching. Fluorescent analysis was performed with a Zeiss Axioplan 2 epifluorescence microscope (Carl Zeiss Microscopy) equipped with fluorescent rhodamine and FITC filters with excitation wavelength ranges of 534-558 nm and 450-490 nm, respectively. Monochrome images were captured by a Dage video camera (Newvicom, Wiesbaden, Germany) and were converted to pseudo-coloured images by Metamorph 6.1 software (Universal Imaging Corporation, Downington, PA, USA).

### Statistics

The statistical significance of differences in means between treatment and control groups were assessed using unpaired t-tests and analysis of variance (ANOVA). P values < 0.05 indicated statistical significance.

## Abbreviations

DRG: dorsal root ganglion; L5: lumbar vertebrae 5; NF-H: non-phospho-specific neurofilament heavy subunit; NF-M: non-phospho-specific neurofilament medium subunit; pNF-H: phosphorylated neurofilament heavy subunit.

## Competing interests

The authors declare that they have no competing interests.

## Authors' contributions

SJ carried out the oxaliplatin and paclitaxel studies and drafted the manuscript. JS carried out the comparison of oxaliplatin and carboplatin. NJ carried out the cisplatin studies. VI provided technical support. BC participated in the design of the study and its coordination. JL and MM conceived of the study, and participated in its design and coordination and drafted the final manuscript. All authors read and approved the final manuscript.
